# An evaluation of factors affecting pain during transrectal ultrasonographic prostate biopsy: a real-life scenario in a retrospective cohort study

**DOI:** 10.7717/peerj.12144

**Published:** 2021-09-06

**Authors:** Oğuz Özden Cebeci, Alp Ozkan

**Affiliations:** 1 Department of Urology, Saglik Bilimleri University, Kocaeli Derince Traning and Research Hospital, Kocaeli, Turkey; 2 Department of Urology, Acıbadem Kocaeli Hospital, Kocaeli, Turkey

**Keywords:** Analgesia, Prostate biopsy, Prostate cancer, Pain, Quality of life

## Abstract

**Background:**

Periprostatic infiltration anesthesia (PPIA) and intrarectal topical anesthesia (IRTA) are recommended methods to control pain in transrectal ultrasonographic prostate biopsy (TRUS-Bx). This study evaluates the factors affecting pain during TRUS-Bx, considering the pathologies involved in anorectal pain etiology and comparing the effectiveness of local anesthesia techniques in providing patient comfort.

**Material and Methods:**

We retrospectively evaluated 477 consecutive patients with TRUS-Bx for elevated Prostate Specific Antigen (PSA), abnormal rectal examination findings, or both. Patients were grouped as local anesthesia methods for pain control during TRUS-Bx. Both groups were compared in terms of age, body mass index, clinical T stage, PSA, prostate volume, number of biopsy cores, type of anesthesia, previous biopsy history, and presence of prostate cancer. We used a visual analog pain scale (VAS) to evaluate the patient’s pain status; pre-procedure (VAS-0), during probe insertion (VAS-I), administration of anesthetic (VAS-A), and simultaneous with the biopsy procedure itself (VAS-Bx). For PPIA and IRTA, 4 ml lidocaine 20 mg/ml injection and 5 g 5% prilocaine-5% lidocaine cream was used, respectively.

**Results:**

The PPIA was used 74.2% (*n* = 354) and IRTA was used for 25.8% (*n* = 123) patients. VAS-0, VAS-I, and VAS-A scores are similar between groups. VAS-Bx was significantly higher in the IRTA than in the PPIA (3.37 ± 0.18 *vs.* 2.36 ± 0.12 *p* > 0.001). Clinical T stage (OR: 0.59), number of biopsy cores (OR: 1.80), and type of anesthesia application (OR: 2.65) were independent variables on TRUS-Bx for pain.

**Conclusion:**

Three factors play roles as independent variables associated with the pain in TRUS-Bx; abnormal rectal examination findings, collection of more than twelve core samples during the biopsy, and the type of anesthesia used. Compared with PPIA, IRTA does not improve pain related to probe insertion, and using IRTA results in higher pain scores for biopsy-related pain. Thus, we recommend a PPIA to lower biopsy-related pain.

## Introduction

Prostate biopsy is considered the gold standard technique for diagnosing prostate cancer (Mottet et al., 2017) and can be performed using either the transrectal or transperineal method (Takenaka et al., 2008). Pain and discomfort are common side effects in both approaches (Rodriguez & Terris, 1998). Anatomical and clinical features such as prostate biopsy history, total prostate volume, and anorectal angle are associated with pain sensation during the biopsy (Sonmez et al., 2020). Patients can feel pain at different stages during the biopsy, as ultrasound probe placement or biopsy sampling, and this pain sensation may continue after the procedure is over (Borghesi et al., 2017). Therefore, administration of periprostatic infiltration anesthesia (PPIA) with or without intrarectal topical anesthesia (IRTA) is used for transrectal ultrasound-guided prostate biopsy (TRUS-Bx) to ensure patient comfort (Mottet et al., 2017; Rappaport et al., 2021; Soloway & Obek, 2000).

Numerous studies in the literature investigate the origin and management of biopsy-related pain (Demirtaş et al., 2020; Kim et al., 2019a; Kim et al., 2019b; Wang et al., 2015). However, daily practice reality is not easily predictable. This study evaluates the factors affecting pain during TRUS-Bx, considering the pathologies involved in anorectal pain etiology and comparing the effectiveness of local anesthesia techniques in providing patient comfort.

## Material and Methods

We retrospectively analyzed 631 patients who underwent TRUS-Bx because of suspected prostate cancer. Prostate biopsy was performed in case of the abnormal digital rectal examination, high serum prostate-specific antigen (PSA) levels, or both. Of these, 154 patients were excluded because of poor anesthesia technique (*n* = 21), having no anesthesia (*n* = 35), missing demographic data (*n* = 59) or an inability to rate a visual analog pain scale (VAS) (*n* = 39). Thus, the database used for analysis consists of 477 patients. Patients were grouped as local anesthesia methods for pain control during TRUS-Bx. Both groups were compared in terms of age, body mass index, clinical T stage, PSA, prostate volume, number of biopsy cores, type of anesthesia, previous biopsy history, and presence of prostate cancer. Study data were collected with the database software FileMaker Pro Version 11. Study data were collected with the database software FileMaker Pro Version 11.

We used GE LOGIQ C series ultrasound (General Electric (GE) Healthcare, Wauwatosa, WI, USA) and a 7.5 MHz E7c-RC transrectal probe for all patients’ biopsy procedures; ciprofloxacin 500 mg was administered 12 h before the biopsy, and rectal cleaning with an enema was performed at least 2 h before the biopsy. Biopsies were performed by two surgeons using the same method and prostate mapping. Liquid vaseline was used for probe lubrication.

**Figure 1:**
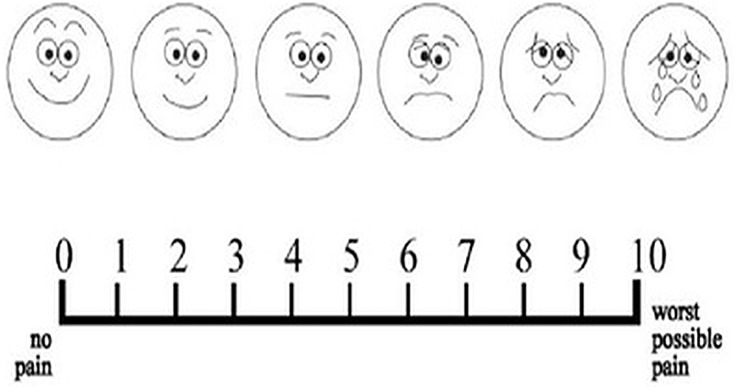
The visual analog scale.

Similar to previous studies, a VAS score using a linear 11-point scale was used to assess the patient’s pain status (Demirtaş et al., 2020) (Fig. 1). We verbally explained the VAS score to the patients before the biopsy, and they were asked to describe VAS#0 as ’no pain or discomfort’ and VAS#10 as ‘the worst possible pain ever experience’. The VAS questionnaire was administered pre-procedure (VAS-0), at probe insertion (VAS-I), an anesthetic application (VAS-A), and simultaneously with the biopsy procedure itself (VAS-Bx).

Before the procedure, patients who had pain in the perianal area were evaluated by history and physical examination to detect anorectal pain etiology, and etiologic factors were recorded.

For the patients receiving PPIA, a 4 ml lidocaine 20 mg/ml injection was performed by applying a 22G, 20 cm Chiba needle to the angle between the base of the prostate and the seminal vesicle on both sides. The biopsy was started at least five minutes following the PPIA injection. For the IRTA group, anesthesia was applied using 5 g of 5% prilocaine-5% lidocaine cream for at least 20 min before the biopsy. Unlike PPIA, the IRTA application was performed before probe insertion.

Patients’ VAS scores were coded in a binary fashion: if VAS-Bx was greater than VAS-I, VAS score was coded as 1; VAS-Bx was less than or equal to VAS-I, VAS score was coded as 0. A new variable was generated to explain pain solely related to the biopsy.

This study was approved by the Saglik Bilimleri University Kocaeli Derince Traning and Research Hospital Ethics Committee (reference: 2020/58). All procedures performed in studies involving human participants followed the ethical standards of the institutional and national research committee and the 1964 Helsinki declaration and its later amendments or comparable ethical standards. Written informed consent was obtained from all individual participants included in the study

### Statistical method

The Stata MP statistical software package (StataCorp, College Station, TX, USA) version 14.2 was used to analyze collected data. The *Shapiro–Wilk normality* test was used to evaluate normal distribution in the data, and a histogram was used to assess homogeneity. The mean ±standard deviation and median (interquartile range [IQR]) were used in the descriptive statistics. The *t*-test for the continuous variables was used when the data met the criteria of a normal distribution. The *Wilcoxon rank-sum* test was used when the data did not meet the criteria of a normal distribution. The *ANOVA* test and the *Spearman* test were used to determine the correlation among the VAS scores. Evaluation of categorical variables was performed by using a *chi-square* test. Univariable and multivariable analyses were performed by the logistic regression method with age, body mass index, clinical T stage, PSA, prostate volume, number of biopsy cores, type of anesthesia, previous biopsy history, and prostate cancer presence. A *p*-value less than 0.2 was set for including variables to construct a model. The final model consists of the clinical T stage, PSA, number of biopsy cores, and type of anesthesia.

## Results

The PPIA method of anesthesia administration was used for 74.2% (*n* = 354) of the study subjects; the IRTA method was 25.8% (*n* = 123). Age, body mass index, PSA, prostate volume, number of biopsy cores, history of prostate biopsy, and presence of prostate cancer were similar among the groups (A detailed evaluation of demographic and clinical data for the PPIA and IRTA groups is shown in Table 1).

**Table 1:** Comparison of the clinical and demographical data between study groups.

** **	**Periprostatic infiltration anaesthesia (*N* = 354)**	**Intrarectal topical anaesthesia (*N* = 123)**	*P*-value
**Age, year;** **mean ± SD**	64.42 ± 7.76	65.32 ± 7.95	.27
**BMI,kg/m** ^**2**^ **; mean ± SD**	27.16 ± 3.68	27.4 ± 4.55	.56
**PSA, (IQR) ng/dL**	7.2 (4.85–12.17)	6.98 (5.11–9.9)	.95
**Prostate volume (IQR) mL**	49.9 (33–74)	50 (35–71)	.96
**Number of cores, %(N)**			
≤12	78.8 (279)	80.5 (99)	
>12	21.2 (75)	19.5 (24)	.79
**Histopathology %(N)**			
Benign	67.5 (239)	69.1 (85)	
Prostate Adenocarcinoma	32.5 (115)	31.7 (38)	.68
**Previous biopsy, %(N)**			
No	68.3 (242)	64.1 (79)	
Yes	31.7 (112)	35.9 (34)	.34
**Clinical T Stage, % (N)**			
T1c	66.2 (234)	64 (91)	
>T1c	33.8 (120)	26 (32)	<0.001
**VAS Pain score**			
VAS pre-biopsy, (Mean ± SD)	0.27 ± 0.05	0.29 ± 0.12	.51
VAS probe insertion, (Mean ± SD)	2.80 ± 0.13	2.39 ± 0.18	.09
VAS anaesthesia, (Mean ± SD)	1.98 ± 0.11	2.18 ± 0.15	.38
VAS biopsy, (Mean ± SD)	2.36 ± 0.12	3.37 ± 0.18	<0.001

**Notes.**

BMI Body Mass Index VAS visual analog scale PSA Prostate-specific antigen SD standard deviation IQR interquartile range

There was no statistically significant difference in VAS-0, VAS-I, and VAS-A scores between the two groups. However, VAS-Bx was statistically significantly higher in the IRTA group than in the PPIA group (*p* < 0.001) (Table 1).

Univariate analysis using age, body mass index, clinical T stage (T1c or ≥T2a), PSA, prostate volume, number of biopsy cores, type of anesthesia, previous biopsy history, and the presence of prostate cancer were used to construct a model. The final model consists of the clinical T stage, PSA, number of biopsy cores, and type of anesthesia. Clinical T stage, number of biopsy cores, and type of anesthesia application were independent variables on TRUS-Bx for pain (Table 2). VAS-Bx in the IRTA group was statistically significantly higher than in the PPIA group (OR: 2.65, 95%; CI [1.71–4.1]) (Table 2).

**Table 2:** Univariable and multivariable regression analyses of factors associated with pain.

**Clinical T Stage**	Univariable, Odds Ratio (95% CI)	p	Multivariable, r Odds Ratio (95% CI)	p
cT1c	reference		reference	
>cT1c	0.62 (0.40–0.95)	0.03	0.59 (0.37–0.96)	.034
**PSA**	1.00 (0.99–1.01)	0.193	1.00 (0.99–1.01)	.166
**Number of cores**				
≤12 cores	reference		reference	
>12 cores	1.82 (1.09–3.04)	0.022	1.80 (1.06–3.05)	.02
**Administration** **method of anaesthesia**				
Periprostatic infiltration anaesthesia,	reference		reference	
Intrarectal topical anaesthesia,	2.7 (1.78–4.17)	<0.001	2.65 (1.71–4.09)	<0.001

**Notes.**

CI, Confidence interval.

**Figure 2:**
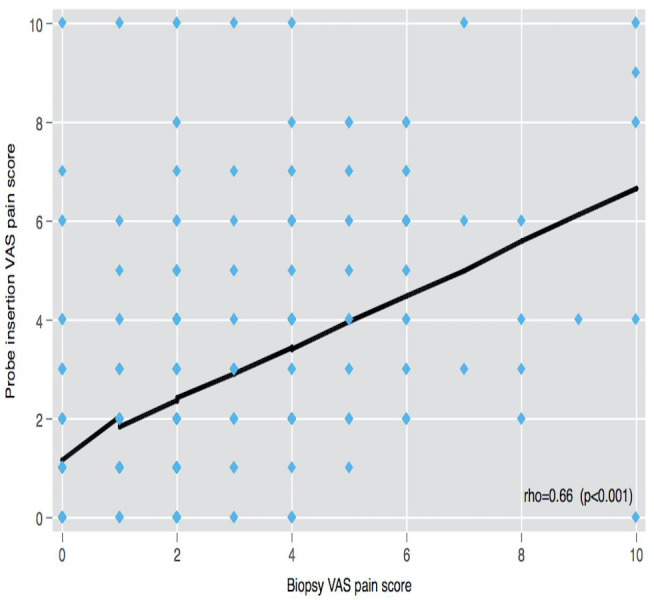
VAS pain scores correlation. VAS: Visual analog scale.

The correlation of VAS-0 with VAS-A, VAS-I, and VAS-Bx scores was low (rho < 0.21), but the linear correlation was found among VAS-A, VAS-I, and VAS-Bx scores (rho > 0.5) (Fig. 2).

In the PPIA group, several instances of other anorectal pathology, such as hemorrhoids (*n* = 17), chronic prostatitis (*n* = 2), and anal fissure (*n* = 1), were found in addition to benign prostatic enlargement (*n* = 43). In the IRTA group, hemorrhoids (*n* = 17), chronic prostatitis (*n* = 3), and inflammatory bowel disease (*n* = 1) in addition to benign prostate enlargement (*n* = 46) were found. Post biopsy, 0.79% (*n* = 5/631) of patients developed urosepsis requiring hospitalization; quinolone-resistant E. coli in blood culture was found in four patients, and E. coli with a broad-spectrum beta-lactam resistance was found in one patient.

## Discussion

Our study revealed that prostate cancer clinical T stage, biopsy core count, and local anesthesia type are independent variables for pain during TRUS-Bx. The VAS-Bx score of the patients in the IRTA group was statistically significantly higher than the PPIA group (OR: 2.65).

TRUS-Bx has been accepted as the gold standard diagnostic method for prostate cancer since it was first described in 1989 (Hodge et al., 1989). A sextant biopsy was used for the definition in this procedure, and no analgesia was prescribed for the initial experience. Nevertheless, 10 or 12 cores performed with an 18G needle and a periprostatic nerve block were defined as standard practice (Mottet et al., 2017). The sensation of discomfort and pain during the biopsy procedure is caused by the insertion of the ultrasonic probe, its manipulation in the rectum, and needle penetration into the prostatic tissue. Previous studies have found a wide range (between 10% and 90%) of patients report discomfort and pain during biopsy procedures (Bastide et al., 2003; Ochiai & Babaian, 2004; Tiong et al., 2007).

PPIA was first reported in a prospective study where 64 patients were randomized into two groups using lidocaine and the other saline injection (Nash et al., 1996). PPIA was performed only unilaterally to the prostate and seminal vesicle junction, described as the prostatic pedicle, and VAS was examined. In the same patients, unilaterally applied PPIA was reported to be less painful than the prostate side without PPIA applied; there was no difference in pain on either side of the prostate in patients using saline.

Soloway et al. reported that with sagittal imaging, injecting approximately 5 ml of 1% lidocaine to both sides of the prostate and the prostate seminal vesicle’s junction to the middle of the prostate and both sides of the prostate at the apex reduced the patient’s discomfort (4). Although pain assessment and analysis of results were not performed using statistical methods, the detailed description of the PPIA technique affected essential changes in the TRUS-Bx procedure’s performance. The use of PPIA became widely accepted after this study.

A meta-analysis found that IRTA reduced pain during probe insertion, but PPIA did not make any difference. Almost all the studies in the meta-analysis were found to have shown increased patient comfort with the simultaneous use of both topical and infiltration anesthesia, a benefit since it has also been reported that a larger number of biopsy cores can be obtained when patients experience less discomfort (Yang et al., 2017). However, in the current study, patients with more than 12 biopsy core samples had more pain (OR: 1.8, 95% CI [1.06–3.05]) regardless of whether IRTA or PPIA was used. We think that this may be due to the pain evaluation method, which used the absolute value difference of pain scales and evaluated them separately before, during, and after the biopsy procedure.

In a randomized prospective, double-blind study, TRUS-Bx was tolerated comfortably without anesthesia (Ingber et al., 2010). The differences in the results of these studies are likely due to differences in their methodology, diverse socio-cultural populations, and factors such as anxiety. Most reported studies excluded patients with pain or comorbidities that cause pain, such as hemorrhoids or anal fissures (Yang et al., 2017). In the current study, we did not exclude these patients from assessing the real-life relationship between TRUS-Bx and pain. However, our results with respect to pain and anesthesia type did not differ from reported studies.

Bastide et al. (2003) reported that, when adjusted for age, prostate volume, number of cores, previous biopsy, initial sampled core, and operator, only the initial sampled core was an independent variable for pain in TRUS-Bx. Since the prostate apex was the most painful site for the initial sampled core, they recommended starting the biopsy procedure from the prostate base.

A recent study reported that patients with large prostate volume, short prostate-anus surface distance, narrow anorectal angle, or biopsy-naive patients might feel relatively more severe pain during the prostate biopsy. The authors stated that these anatomical features could be calculated with multiparametric magnetic resonance imaging (mpMRI) before the biopsy (Sonmez et al., 2020).

Some conflicting studies suggest that topical anesthesia is ineffective against a placebo or that topical anesthesia is more effective than a placebo (Cevik et al., 2002; Issa et al., 2000; Rodriguez et al., 2003). However, in the design of these studies, topical anesthesia is not analyzed separately for its effect, as is the anesthesia method, drug, dose, and duration of application. Due to these findings, it is difficult to reach a definite conclusion about topical anesthesia. Furthermore, most studies on this subject do not provide clear information on the factors that may effectively affect anorectal pain etiology, thus not reflecting real-life circumstances. In the current study, patients with pain before biopsy were evaluated for a differential diagnosis that could play a role in the etiology of anorectal pain. As a result, we found that 18.6% (*n* = 89/477) of the patients had pathologies that caused anorectal pain. When the PPIA and IRTA groups were compared, there was no statistically significant difference between VAS-0, VAS-I, and VAS-A scores. The lack of correlation of VAS-0 with other VAS scores in these patients (rho < 0.21) reveals the importance of evaluating other pathologies in the etiology of anorectal pain. We believe that IRTA has either a positive or a negative effect on pain because the factors involved in the pain etiology were not examined in the previously reported studies.

A meta-analysis reported that using PPIA and IRTA simultaneously lowered the VAS score for probe insertion, anesthesia, and biopsy. A subgroup analysis said that any local anesthetic agent provides better pain control than sedation or non-steroidal anti-inflammatory drugs (Wang et al., 2015). In our study, there was no group with a combined anesthetic application. However, there was no difference in the VAS-0 scores of the patients who underwent anesthesia before the procedure; only the VAS-Bx score was statistically significantly higher in the PPIA group than in the IRTA group (OR: 2.65, 95% CI [1.71–4.1]): (Table 2). Thus the VAS-I score—that is, the reported pain level at the time of probe insertion—was not improved with IRTA. For this reason, our study does not support the simultaneous use of IRTA with PPIA to lower the pain associated with probe insertion.

In the current study, minor complications were not reported, although the post-biopsy infection rate was similar to previously reported results in a similar population (Avcıet al., 2018; Efesoy et al., 2013).

The retrospective and non-randomized design is the most important restriction of this study. Other limitations include that two different surgeons performed the biopsies, the absence of using mpMRI and that the socio-cultural demographics of the patients studied were not known.

## Conclusions

Three factors play roles as independent variables associated with the pain in TRUS-Bx: abnormal rectal examination findings, collection of more than twelve core samples during the biopsy, and the type of anesthesia used. Anesthesia administration, probe insertion, and biopsy VAS scores correlated poorly with pre-biopsy VAS scores. However, the probe insertion VAS score correlates well with the biopsy VAS score. We conclude that, compared with PPIA, IRTA does not improve pain related to probe insertion, and using IRTA results in higher pain scores for biopsy-related pain. Thus, we recommend a PPIA to lower biopsy-related pain.

##  Supplemental Information

10.7717/peerj.12144/supp-1 Supplemental Information 1 Patient demographic and clinical raw data

## Additional Information and Declarations

### Data Availability

The following information was supplied regarding data availability: The raw data is available in the Supplementary File.
